# Why Angiotensin II is a Poor Choice for Circulatory Support of Ventilated COVID-19 Patients Compared to Vasopressin

**Published:** 2022-09-20

**Authors:** Robert C. Speth, Michael Bader

**Affiliations:** 1 Department of Pharmaceutical Sciences, College of Pharmacy, Nova Southeastern University, Fort Lauderdale, FL 33328 USA; 2 Department of Pharmacology and Physiology, School of Medicine, Georgetown University, Washington, DC, 20057 USA; 3 Max-Delbrück-Center for Molecular Medicine (MDC), Robert-Rössle-Str.10, D-13125 Berlin, Germany; 4 German Center for Cardiovascular Research (DZHK), Partner Site Berlin, Hessische Strasse 3-4, D-10115 Berlin, Germany; 5 Charité Universitätsmedizin Berlin, corporate member of Freie Universität Berlin and Humboldt-Universität zu Berlin, Charitéplatz 1, D-10117 Berlin, Germany; 6 University of Lübeck, Institute for Biology, Ratzeburger Allee 160, D-23562 Lübeck, Germany

## Abstract

Early in the COVID-19 pandemic when it was first reported that SARS-CoV-2 used membrane-bound angiotensin-converting enzyme-2 (ACE2) as its receptor for entry into cells, warnings were raised against the use of angiotensin-converting enzyme (ACE) inhibitors and angiotensin receptor blockers (ARBs) because of their potential to increase ACE2 expression. These reports ignored the adverse effects that the renin-angiotensin system (RAS) exerts on the cardiovascular system and kidneys via its primary hormone angiotensin (Ang) II acting upon AT_1_ receptors that could exacerbate the cytokine storm induced by SARS-CoV-2 ^1^. At one point it was even recommended that COVID-19 patients suffering from cardiovascular collapse be administered Ang II to restore blood pressure rather than norepinephrine or vasopressin ^2^. An alternative strategy for treating COVID-19 was the administration of soluble ACE2 (sACE2) to act as a decoy receptor for the virus, misdirecting it away from vulnerable cells expressing membrane bound ACE2 ^3–5^. However, a paper published in early 2021 ^6^ described a scenario in which sACE2 and vasopressin played essential roles in SARS-CoV-2 infection of cells vulnerable to the virus. This commentary challenges both the ^2^ and ^6^ reports based upon their misconceptions and technical errors that pose a threat to the administration of life-saving therapies for severely affected COVID-19 patients.

## Introduction

The SARS corona virus (CoV) cast the renin-angiotensin system (RAS) into the spotlight of infectious disease mechanisms when angiotensin-converting enzyme-2 (ACE2) was found to be the “receptor” by which the SARS-CoV infected cells ^7^. While not part of the mainstream RAS, angiotensin-converting enzyme-2 (ACE2) functions as a component of the “counter-regulatory” RAS, known as the ACE2/Ang 1–7/Mas axis, as recently reviewed ^8^. Thus, while the classical RAS, also known as the ACE/Ang II/AT_1_R axis causes a number of pathophysiologies, especially inflammation, see reviews ^9, 10^, ACE2 by degrading angiotensin (Ang) II to Ang 1–7 reduces the pro-inflammatory actions of the classical RAS while generating a peptide that exerts anti-inflammatory effects ^11, 12^. Early into the COVID-19 pandemic ACE2 was again shown to be the “receptor” for SARS-CoV-2 ^13–15^. Indeed, one of the mechanisms contributing to the SARS-CoV-2 cytokine storm mediated inflammation damage in COVID-19 is by decreasing ACE2 expression, thereby increasing Ang II levels ^16, 17^.

## Objective

The objective of this commentary/perspective is to critically review recent publications by Busse et al., 2020 ^2^ and Yeung et al., 2021 ^6^ to refute the inaccurate conclusions reached by these authors that are contradictory to proper therapeutic treatment of COVID-19.

Given the contribution of Ang II to COVID-19 pathology, the suggestion that Ang II be used as a pressor agent to treat hypotensive shock in COVID-19 patients ^2, 18^ must be challenged ^1^. In addition to the proinflammatory actions of Ang II that can exacerbate the cytokine storm-induced damage, an adverse side effect arising from the use of Ang II as a vasopressor is formation of blood clots https://www.fda.gov/news-events/press-announcements/fda-approves-drug-treat-dangerously-low-blood-pressure#:~:text=The%20U.S.%20Food%20and%20Drug,septic%20or%20other%20distributive%20shock (accessed July 20, 2022), also characterized as deep vein thromboses ^19^. As venous thrombosis formation is one of the major toxicities arising from COVID-19 ^20, 21^ this is yet another basis for not treating COVID-19 patients with Ang II.

## Angiotensin II as a pressor agent: Comparison with other pressor agents

Giapreza®, Ang II was approved for use in vasopressor treatment-resistant individuals based upon greater elevation in mean arterial pressure after 3 hours treatment and reduced sequential organ failure assessment (SOFA) scores at 48 hours compared to placebo treatment in a population of patients, 80.7%, suffering septic shock ^19^. While there was a trend towards greater 28-day survival in the Ang II treated group, it was not significantly different from the placebo group. An acknowledged limitation of this study was the failure to assess long-term outcomes between groups ^19^. Known adverse effects of Ang II include nephrotoxicity ^10, 22^ and endothelial dysfunction ^23^ which may not be manifested as impaired functionality within a 28-day time frame. Additionally, phenylephrine, a selective alpha_1_ adrenergic agonist that can be used as a pressor agent ^24^ was not assessed in a comparison group for the Ang II pressor therapy group.

While it is beyond the scope of this commentary to critically evaluate different potential therapies for the treatment of norepinephrine and vasopressin resistant hypotension with septic shock, other options include addition of a beta_1_ adrenergic receptor blocker such as esmolol to decrease the tachycardic effects of norepinephrine ^25^. As noted above, phenylephrine is a pressor agent that could elevate blood pressure without the adverse effects on the heart or decreased blood flow to the jejunal region associated with the beta_1_ adrenergic agonistic effects of norepinephrine ^24^.

## Soluble ACE2 (sACE2) and SARS-CoV

While there are now effective vaccines against infection with the early strains of SARS-CoV-2, prior to their development one of the strategies devised for treating Acute Respiratory Distress Syndrome (ARDS) associated with the SARS-CoV-1 was administration of a recombinant ACE2 ^12, 26^. This approach was resurrected for treatment of COVID-19 ^3–5^, primarily to serve as a decoy receptor for SARS-CoV-2 competing for membrane-bound ACE2 on vulnerable cells. A secondary benefit of administration of soluble ACE2 (sACE2) is that it could reduce the level of Ang II available to produce proinflammatory responses via the AT_1_ receptor while generating Ang 1–7 that reportedly activates Mas receptor-mediated anti-inflammatory responses ^27^.

Given this rationale for administering sACE2 as a therapeutic for COVID-19, a paper published in the spring of 2021 made the surprising claim that SARS-CoV-2 infection of cells requires sACE2 ^6^. The authors proposed that vasopressin bound to SARS-CoV-2 mediates the uptake of the virus into cells after binding to sACE2, whereupon it binds to the AVPR1B receptor on cell membranes, which then internalizes into cells with its SARS-CoV-2/ACE2/vasopressin cargo. Strangely, they used not the nonapeptide vasopressin, but its 15 kDa precursor in their experiments, which should not bind to this receptor. Moreover, neither the precursor nor the AVPR1B receptor for vasopressin are expressed in lung cells relevant for SARS-CoV-2 infection. They additionally propose an alternative route of infection in which sACE2 bound to SARS-CoV-2 engages with AT_1_ angiotensin II receptors leading to AT_1_ receptor mediated endocytosis of the SARS-CoV-2/sACE2 complex. However, there is no evidence that extracellular ACE2 can bind to the extracellular domains of the AT_1_ receptor.

## Adverse consequences of inaccurately implicating vasopressin as a component of the COVID-19 infection process

A serious concern regarding these invaliding errors and inconsistencies of this paper is that it can adversely affect therapeutic approaches to the treatment of COVID-19. Already the Yeung et al. study has spawned one clinical study that was carried out based upon the potential adverse effects of the use of vasopressin for treatment of COVID-19 patients ^28^. This study showed that vasopressin infusion is not associated with impaired viral clearance, so that it can continue to be used safely as a pressor agent for circulatory support for COVID-19-patients needing mechanical ventilation. This study should not have had to be done, with the potential for Ang II being used for circulatory support instead of vasopressin, which causes less pulmonary artery vasoconstriction than norepinephrine or Ang II ^29^. Of further note, Battle et al., 2022 ^30^ have recreated the experimental protocol of Yeung et al., 2021 ^6^ and failed to replicate their results.

## Conclusions

The COVID-19 pandemic has disrupted lives and economies worldwide creating an urgency to develop treatments to mitigate this disease. The involvement of the RAS in this disease, with ACE2 acting as the receptor for SARS-CoV-2 has led to conceptualization of therapeutic approaches by which membrane bound ACE2 expression is inhibited, formation of Ang II is inhibited by administration of ACE inhibitors, blockade of AT_1_ angiotensin II receptor function, and development of soluble ACE2 to act as a decoy receptor for the virus have all been proposed. Given the pathophysiological actions of Ang II that promote inflammation and constrict pulmonary blood flow, Ang II is a poor choice for maintenance of blood pressure in ventilated COVID-19 patients. The regrettable paper by Yeung et al., 2021 falsely implicating vasopressin binding to AVPR1B receptors as the mechanism for SARS-CoV-2 infection of cells would suggest that vasopressin not be used for circulatory support in ventilated patients, despite its advantage over norepinephrine and Ang II by virtue of its reduced pulmonary artery constriction, thereby protecting blood flow to the lungs. The failure to correct or retract this publication can only adversely affect the treatment of severely ill COVID-19 patients.
